# An annotated dataset of images of Chinese giant salamanders

**DOI:** 10.1016/j.dib.2026.112552

**Published:** 2026-02-06

**Authors:** Xinyao Yang, Junyi Chen, Didi Lu, Nanqing Sun, Mokai Xie, Haotian Qian

**Affiliations:** a College of Intelligent Systems Science and Engineering, Hubei Minzu University, Enshi 445600, China; b School of Food and Strategic Reserves, Henan University of Technology, Zhengzhou 450000, China

**Keywords:** Chinese giant salamander, Deep learning, Object detection, Computer vision

## Abstract

The Chinese giant salamander is classified as a Class II protected species in China and is recognized as critically endangered by the International Union for Conservation of Nature (IUCN). Due to their unique behavioral patterns, wild Chinese giant salamanders are primarily nocturnal and inhabit areas characterized by complex terrain, which results in limited detection coverage and significant challenges in observation. Consequently, images of wild Chinese giant salamanders are exceedingly rare, and the scarcity of existing data impedes the advancement and application of deep learning-based object detection models. This study constructs and releases a specialized dataset for Chinese giant salamanders, comprising 1386 images and a total of 1397 annotated bounding boxes. All images represent diverse field scenarios and are meticulously annotated in accordance with YOLO (You Only Look Once) labeling specifications. Annotation files are provided in both PASCAL VOC (Visual Object Classes) and COCO (Common Objects in Context) formats to ensure compatibility with leading detection frameworks, including YOLO v8 and YOLO v11. This dataset aims to offer high-quality, multi-scenario annotated data for research in computer vision and conservation biology, facilitating the training and evaluation of models for intelligent monitoring and species conservation of the Chinese giant salamander, thereby promoting the development of visual recognition technologies for endangered species.

Specifications Table

**Table:** 

Subject	Computer Sciences
Specific subject area	An annotated dataset of images of Chinese giant salamanders.
Type of data	image (jpg, png) and corresponding annotation file
Data collection	*The image is a video extraction frame taken using an EOS RP (Canon Corporation, Tokyo, Japan) camera and a surveillance camera of the TL-IPC642-A (Pulian Technology Co., Ltd., Shenzhen, China) model. Each image of this dataset contains at least one Chinese giant salamander, and there are 1386 images and 1397 annotated bounding boxes in the entire dataset.*
Data source location	*Xianfeng County, Hubei Province, China*
Data accessibility	Repository name: Mendeley Data Data identification number: 10.17632/xzvdkhr4bg.1 Direct URL to data: https://data.mendeley.com/datasets/xzvdkhr4bg/1

## 1 Value of the Data

• The dataset provides high-quality, well-labeled data, which helps train better Chinese giant salamander detection models.
• The dataset presented includes photographs of Chinese giant salamanders taken in various complex conditions in the wild. This dataset can be used for a variety of applications, such as image processing, image segmentation, machine learning, and deep learning, for detecting Chinese giant salamanders in the wild.
• The dataset contains annotated images of Chinese giant salamanders, providing a valuable resource for developing and refining machine learning models for application in classification and regression. The multiple standardized formats allow for easy integration of datasets into object detection frameworks such as YOLO v8, YOLO v11, and more, supporting the training and application of compatible models and facilitating research expansion and innovation.
• Object detection models trained using this dataset can be integrated into web applications, invoked within custom-developed software, or deployed on edge hardware after model lightweighting. All these deployment methods enable real-time detection of Chinese giant salamanders or large-scale image detection of Chinese giant salamanders.
• This dataset can facilitate the work of researchers for the research community to work in computer vision, research related to Chinese giant salamanders, and conservation bases related to Chinese giant salamanders.

## 2 Background

Computer vision has emerged as a transformative technology in the field of animal identification. However, the utilization of deep learning algorithms in these tasks relies on the availability of robust datasets. Despite the growing interest in applying computer vision to animal identification, data related to the Chinese giant salamander is still relatively scarce [1,2]. The scarcity of datasets severely hinders the refinement and advancement of deep learning models tailored to address the unique challenges of diverse environments. To address this issue, we have created a specialized dataset comprising 1386 annotated images of Chinese giant salamanders. This dataset serves as a foundational resource for developing sophisticated computer vision applications dedicated to Chinese giant salamander recognition. It enables the training of deep learning models to accurately identify these salamanders across diverse environments, thereby contributing to species recovery efforts. This dataset is expected to advance computer vision applications related to the Chinese giant salamander, particularly within the field of endangered species conservation. It will empower researchers and practitioners to develop algorithms that are not only precise but also adaptable to real-world complexities. Furthermore, by providing a standardized benchmark, the dataset will foster innovation in evaluating the performance of computer vision systems within intricate environments.

## 3 Data Description

**Fig. 1:**
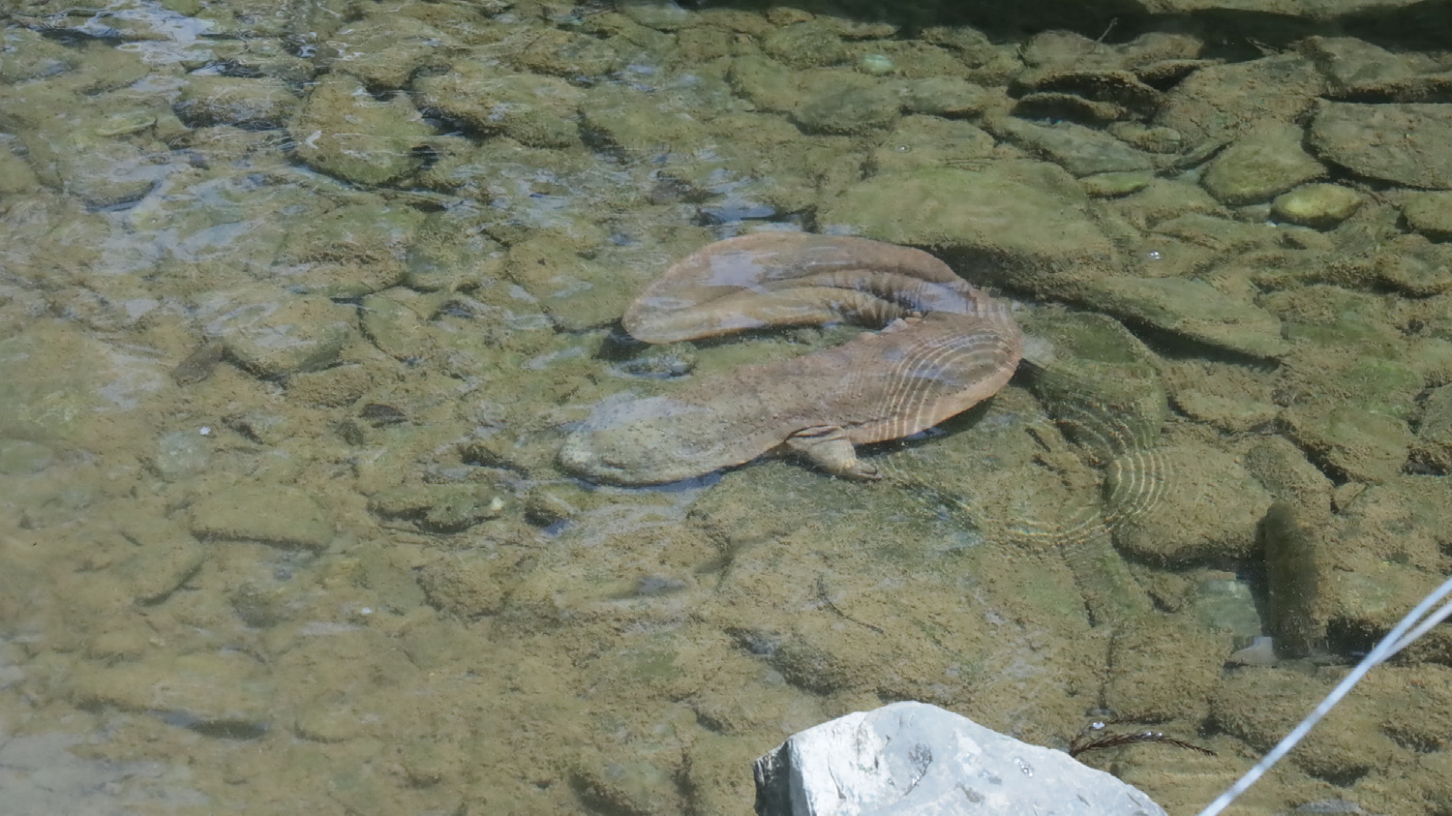
Original camera image.

This dataset of Chinese giant salamanders aims to address existing challenges in identification systems for Chinese giant salamanders, particularly those related to rapid identification in the field and other factors that may affect accuracy. Fig. 1 below shows the original camera image.

**Fig. 2:**
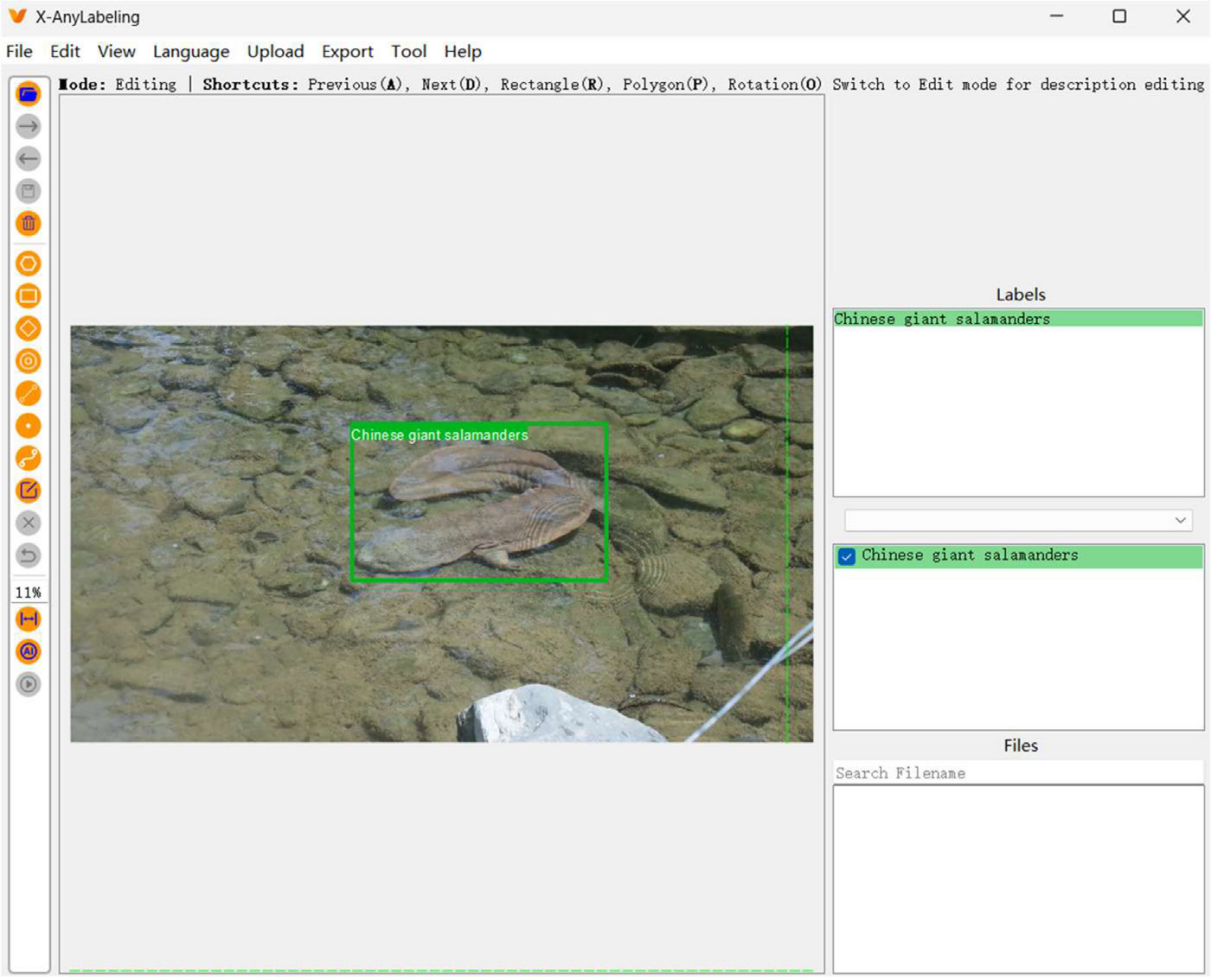
Annotation process.

The image is processed in a way that maintains its original size during the marking phase. We use the open-source X-AnyLabeling [3]. When labeling images, we ensure that each bounding box accurately covers the entire Chinese giant salamander, minimizing unnecessary background pixels, as shown in Fig. 2. The chosen annotation format is widely used in popular object detection environments, providing researchers with convenient tools to easily integrate proposed datasets into their work. This simplifies the training process for object detection models, eliminating the need to convert annotation files into various formats.

**Fig. 3:**
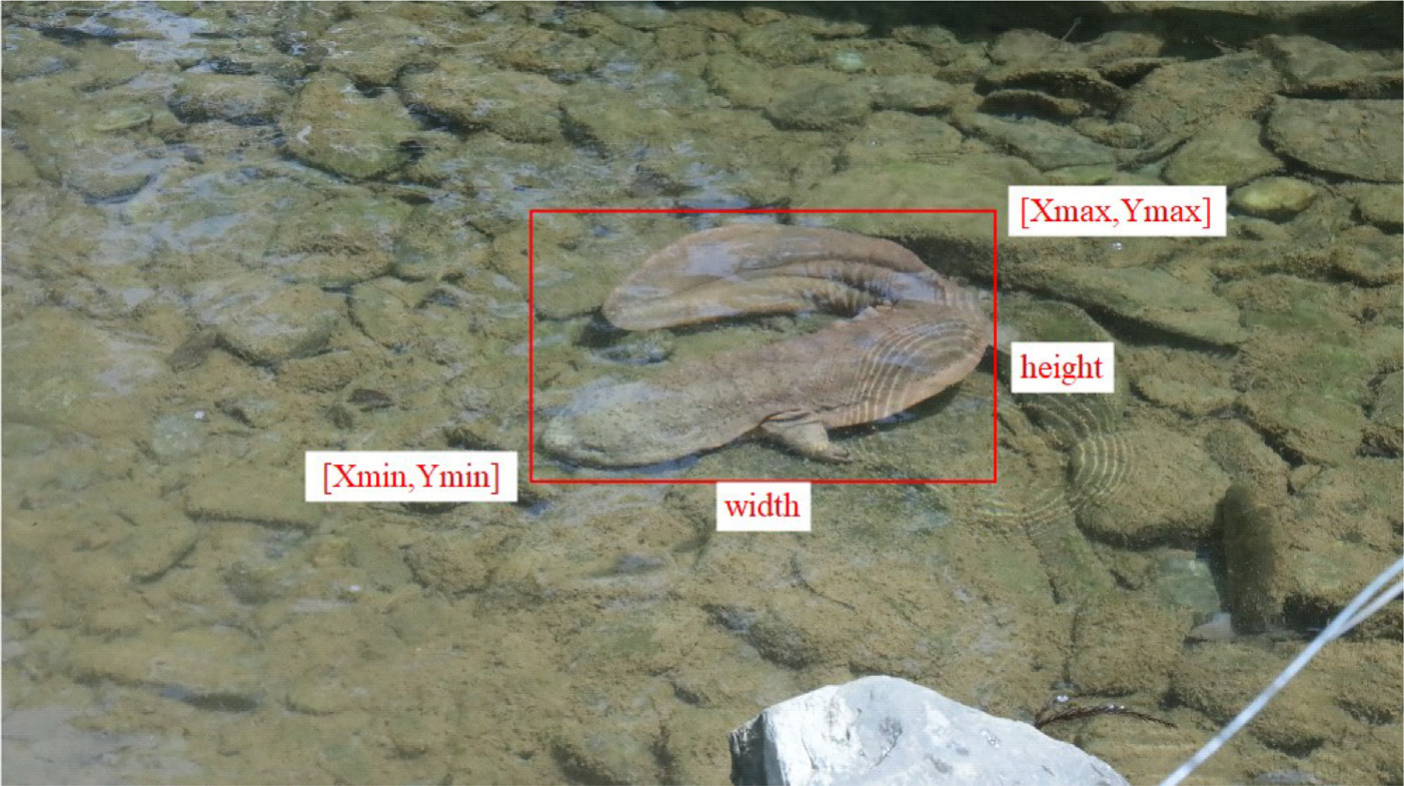
Pascal VOC annotation format.

The Pascal VOC (Visual Object Classes) [4] format is an XML (Extensible Markup Language) file annotation containing the coordinate information of the Chinese giant salamander target box, Xmin, Ymin, Xmax, Ymax. Based on this information, the height and width of the bounding box can be calculated. The Pascal VOC annotation format is shown in Fig. 3 below.

**Fig. 4:**
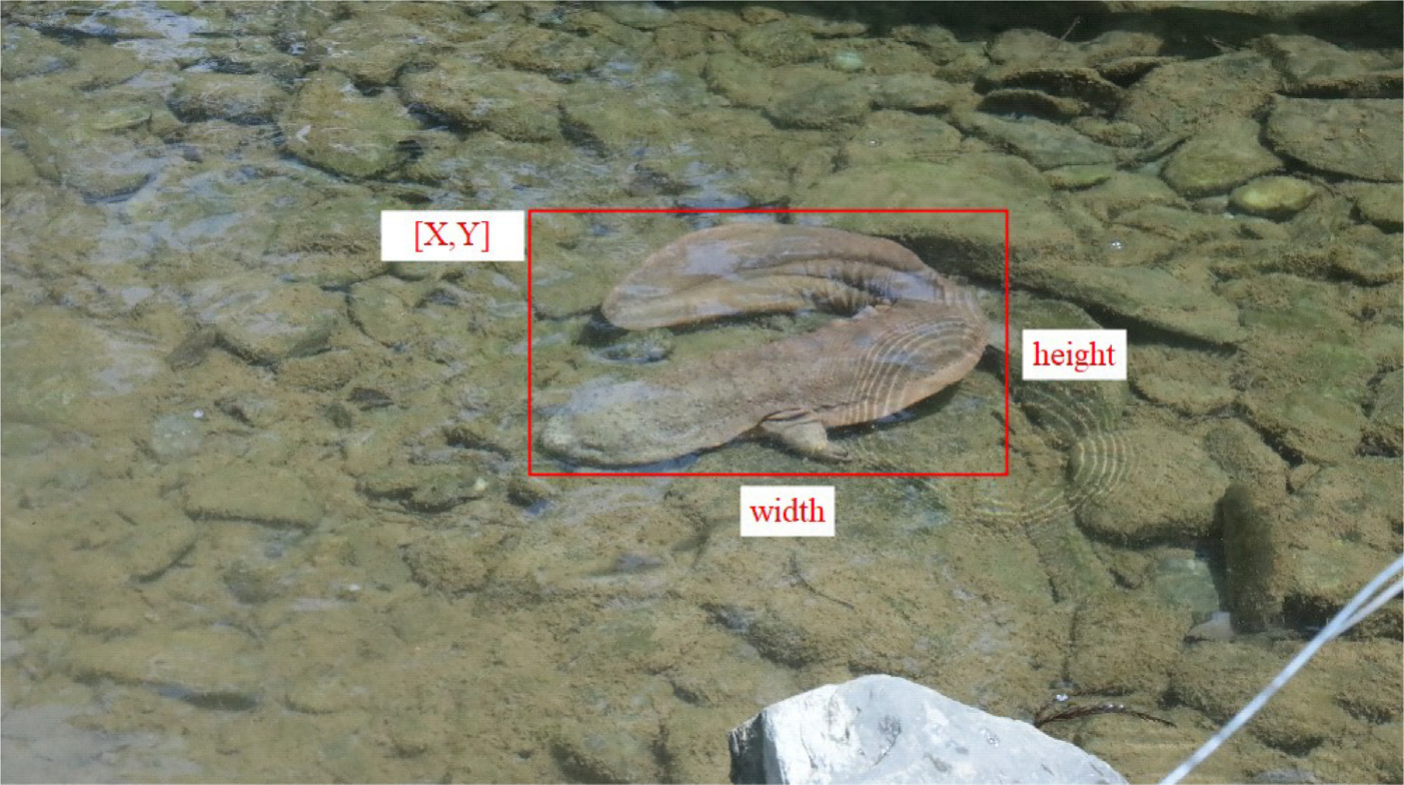
COCO annotation format.

The COCO (Common Objects in Context) [5] format is widely adopted as the standard data format for training and inference in object detection tasks, requiring all data related to object detection tasks to conform to the COCO format. The COCO format is a JSON (JavaScript Object Notation) structure that specifies the formatting of dataset labels and metadata, and it is one of the most popular datasets in object detection. The JSON file contains the x and y coordinates of the target box of the Chinese giant salamander, as well as their height and width, and the COCO annotation format is shown in Fig. 4 below.

**Fig. 5:**
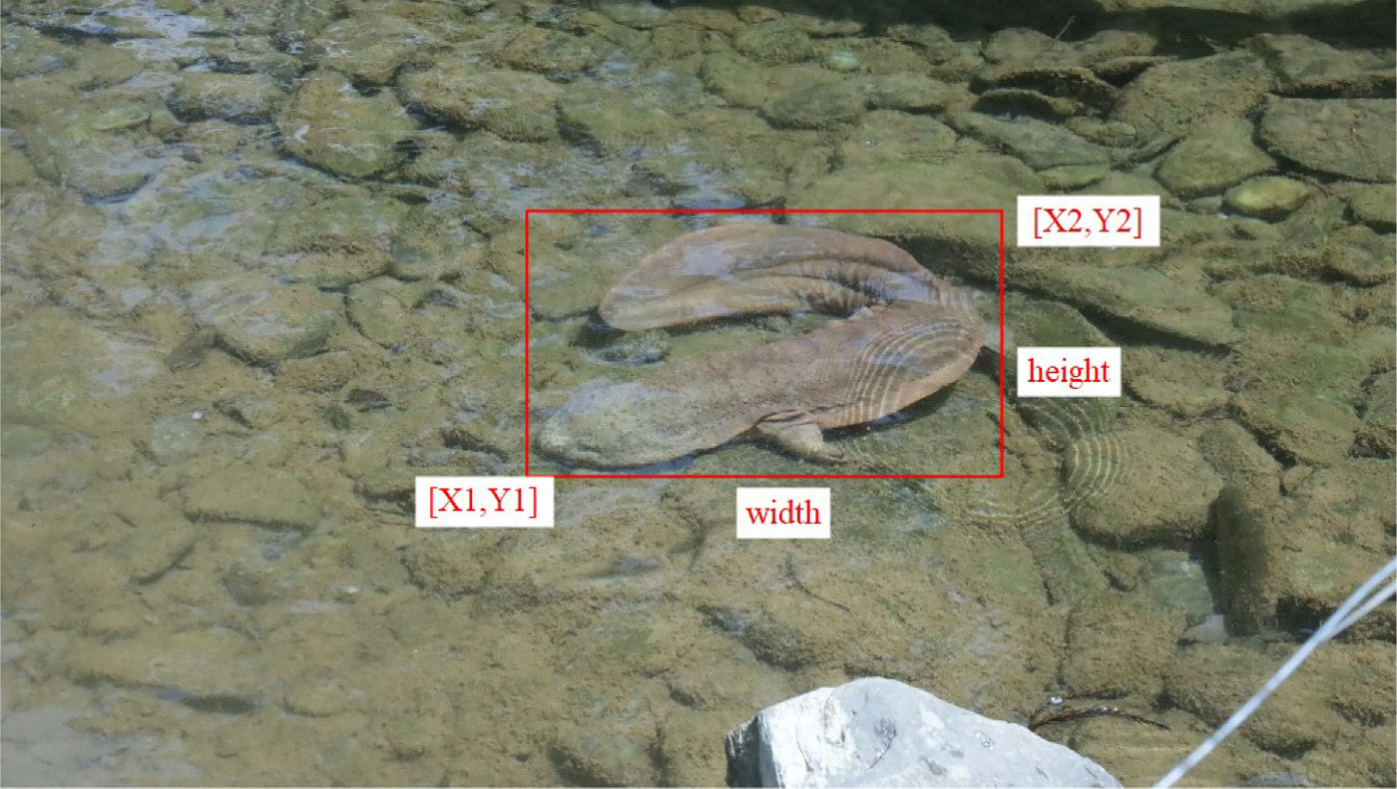
TXT annotation format.

The YOLO [6] series of networks contains a TXT (Text File Format) file per image containing annotations and numeric representations of labels, as well as a label mapping that maps numeric IDs to human-readable strings. Annotations are normalized to be in the range of [0,1], making them easy to process even after scaling or stretching the image. The format is popular for its high compatibility with framework implementations for various YOLO models, and the TXT annotation format is shown in Fig. 5 below.

The collected and processed data are organized into three separate folders: raw images, annotation files, and labeled images of Chinese giant salamanders for training. A brief description of the dataset files is shown in Table 1.

**Table 1:** Brief description of the dataset file.

No.	Name	Type/Format	Description	Size
1	Full dataset	Root folder	Easily packaged for download	3.06GB
2	Original image	Compressed (.zip) folder: 1386 JPG images	Original camera image, containing many images of Chinese giant salamanders	941MB
3	Annotation file	Three subfolders: JSON, XML, and TXT, for single-class annotation files for all images	Annotate the original image with X-AnyLabeling and label it as “Chinese giant salamander” in the annotation file in a different format	1.23GB
4	Image of a Chinese giant salamander marked for training	The root folder contains three subfolders: image, label, Original image, and 1386 JPG images are included in “Original image”	According to the ratio of 7:2:1, 1386 JPG format images are divided into “train”, “test”, and “val” for easy training	941MB

### 3.1 Original images

This folder contains 1386 images in JPG format. The images were taken from videos taken by two cameras, and each image features a Chinese giant salamander.

### 3.2 Labeling images of Chinese giant salamanders used for training

This folder contains 3 subfolders, dividing 1386 JPG images into “train”, “test”, and “val” according to a 7:2:1 ratio for easy training.

### 3.3 Annotation documents

The folder contains three subfolders, each containing annotation files for all the original images in JSON, XML, and TXT formats. These files are labeled as 'Chinese giant salamander' using X-AnyLabeling software.

### 3.4 Image types

This dataset comprises 1386 images in JPG format, each containing at least one Chinese giant salamander. Among these, 30 images were captured indoors, while 1356 were taken outdoors. All indoor images feature ample lighting. The outdoor images include 572 images under normal daylight conditions and 784 images under low-light conditions. Table 2 presents the data distribution.

**Table 2:** Distribution of images in the chinese giant salamander dataset under different shooting environments and lighting conditions.

Shooting Environment	Lighting conditions	Number (pieces)	proportion
Indoor Environment	Normal lighting	30	2.16 %
Outdoor environment	Normal daylight illumination	572	41.27 %
	Low-light environment	784	56.57 %
Total		1386	100 %

## 4 Experimental Design, Materials and Methods

**Fig. 6:**
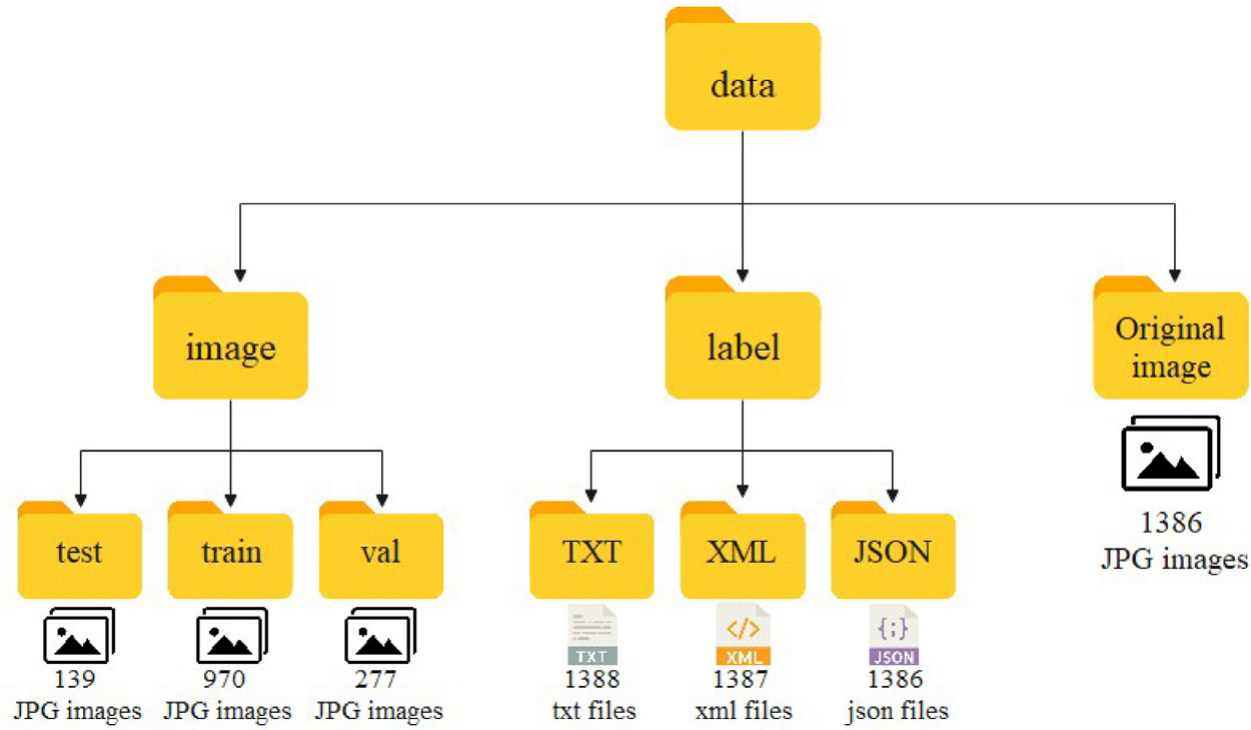
Dataset structure.

Fig. 6 shows the structure and construction method of our annotated Chinese giant salamander image dataset and its environmental background.

The original footage was captured using two TL-IPC642-A surveillance cameras and one EOS RP camera, positioned within the Zhongjianhe Chinese Giant Salamander National Nature Reserve in Xianfeng County, Hubei Province, China. The filming period extended from March to December 2025. Given the nocturnal habits of the Chinese giant salamander and its status as a rare species, four to five video segments were selected from the recorded activity periods. To ensure a diverse representation of both the salamander's movement patterns and environmental backgrounds, one frame per second was extracted, thereby enriching the variety of the dataset.

In this study, we employed the professional image annotation tool X-AnyLabeling for manual annotation. Prior to formal annotation, we established standardized annotation guidelines for Chinese giant salamanders for all annotators. These guidelines explicitly defined annotation targets as exclusively adult and subadult Chinese giant salamanders, excluding background distractors, with tightly fitted bounding boxes. All annotators underwent standardized training and practiced using a set of 20 standard test images until their annotations achieved high consistency with expert standards.

To ensure annotation accuracy and consistency, we executed a five-round annotation validation process across all 1386 images. The first round involved two trained annotators independently performing bounding box annotations on all images, generating two independent sets of primary annotation files. The second round involved two additional annotators reviewing the data to ensure all Chinese giant salamanders were annotated while excluding other environmental objects. The third round performed cross-validation and disagreement detection by comparing annotations (including target count, position, and size) across the two datasets via Python scripts, automatically flagging images with discrepancies. A discrepancy is defined as either: Discrepancies in the total number of detected targets across the two annotators for the same image, or Any target with a bounding box intersection ratio below 0.85. The fourth round involves expert arbitration. All images with discrepancies undergo final review by two senior annotators with over five years of annotation experience. Arbitrators refer to the original image and both independent annotations to determine the correct target count and bounding box locations, making the final ruling. In the fifth round, based on the arbitration results, we generated a single, verified final annotation set. Subsequently, we randomly sampled 10 % of the annotations for final quality review by expert arbitrators, confirming that all bounding boxes complied with specifications and no systematic errors were found.

We convert these rigorously quality-controlled annotations into annotation files across three widely adopted formats (XML, JSON, and TXT), commonly used in object detection. Compatible with YOLO networks, COCO, and PASCAL VOC formats, these standardized, ready-to-use annotation files significantly lower the technical barrier for utilizing this dataset. Researchers can seamlessly train, validate, and compare different algorithms and models using this dataset without cumbersome and potentially biased format conversions. This ensures that studies based on this dataset can be accurately and efficiently reproduced and compared.

This dataset serves as a valuable resource for researchers dedicated to developing high-performance machine learning models for intelligent detection of Chinese giant salamanders. Beyond direct applications, the dataset holds exploration potential across multiple fields including computer vision, precision forestry, robotics, and broader wildlife conservation research. In summary, this project can make significant contributions to advancing research and technology related to Chinese giant salamanders.

## Limitations

Although the data were collected from specific river sections in Xianfeng County, Hubei Province, China, and cover complex conditions in the wild, they may not fully represent the morphology and environmental characteristics of Chinese giant salamanders in other regions or under varying water quality, light, and seasonal conditions. This limitation may affect the model's generalizability in different contexts. The current dataset provides bounding box annotations in three formats: YOLO, COCO, and PASCAL VOC. In the future, it may be beneficial to consider multi-level annotations, such as key points, segmentation masks, or behavioral attributes, to support more fine-grained visual analysis tasks.

## Ethics Statement

Throughout the construction of this paper, the author generally adheres to the ethical standards expected in scientific publishing. The data collection for this work employed non-invasive observation and monitoring of wildlife, during which no harm was inflicted upon any Chinese giant salamander, thus complying with research ethics norms.

## CRediT authorship contribution statement

**Xinyao Yang:** Writing – original draft, Software, Data curation, Investigation. **Junyi Chen:** Writing – review & editing, Validation, Resources, Project administration, Methodology, Conceptualization, Funding acquisition. **Didi Lu:** Writing – review & editing, Validation, Resources, Conceptualization. **Nanqing Sun:** Software, Data curation, Investigation. **Mokai Xie:** Validation, Methodology, Conceptualization. **Haotian Qian:** Validation, Methodology, Conceptualization.

## Data Availability

Mendeley DataAn annotated dataset of images of Chinese giant salamanders (Original data)
